# Untargeted metagenomics shows a reliable performance for synchronous detection of parasites

**DOI:** 10.1007/s00436-020-06754-9

**Published:** 2020-06-26

**Authors:** Claudia Wylezich, Simone M. Caccio, Julia Walochnik, Martin Beer, Dirk Höper

**Affiliations:** 1 Institute of Diagnostic Virology, Friedrich-Loeffler-Institut, Federal Research Institute for Animal Health, Südufer 10, 17493 Greifswald-Insel Riems, Germany; 2 Department of Infectious Diseases, Istituto Superiore di Sanità, Viale Regina Elena 299, 00161 Rome, Italy; 3 Molecular Parasitology, Institute for Specific Prophylaxis and Tropical Medicine, Medical University of Vienna, Kinderspitalgasse 15, 1090 Vienna, Austria

**Keywords:** Metagenomics, Parasite detection, Ribosomal RNA, Reference mapping, Sequence type, Proficiency test

## Abstract

Shotgun metagenomics with high-throughput sequencing (HTS) techniques is increasingly used for pathogen identification and characterization. While many studies apply targeted amplicon sequencing, here we used untargeted metagenomics to simultaneously identify protists and helminths in pre-diagnosed faecal and tissue samples. The approach starts from RNA and operates without an amplification step, therefore allowing the detection of all eukaryotes, including pathogens, since it circumvents the bias typically observed in amplicon-based HTS approaches. The generated metagenomics datasets were analysed using the RIEMS tool for initial taxonomic read assignment. Mapping analyses against ribosomal reference sequences were subsequently applied to extract 18S rRNA sequences abundantly present in the sequence datasets. The original diagnosis, which was based on microscopy and/or PCR, could be confirmed in nearly all cases using ribosomal RNA metagenomics. In addition to the pre-diagnosed taxa, we detected other intestinal eukaryotic parasites of uncertain pathogenicity (of the genera *Dientamoeba, Entamoeba, Endolimax, Hymenolepis*) that are often excluded from routine diagnostic protocols. The study clearly demonstrates the applicability of untargeted RNA metagenomics for the parallel detection of parasites.

**Electronic supplementary material:**

The online version of this article (10.1007/s00436-020-06754-9) contains supplementary material, which is available to authorized users.

## Introduction

Microscopy remains the gold standard for diagnosis of many parasitic infections, but requires skilled personnel and is time consuming. Moreover, morphologic features are often insufficient to identify the organism at the species level. Furthermore, the relatively low prevalence of many parasitic infections in Western industrialized countries has caused a decrease in awareness and a loss of expertise. Conventional molecular pathogen detection is also a challenge for emerging or neglected pathogens for which no sequence information and consequently no well-established test systems are available. The establishment of new specific tests is complicated by the lack of standardized reference material, especially in the case of parasites. For all these reasons, a fast and efficient one-serves-all approach is desirable for rapid and synchronous parasite detection.

Untargeted (PCR-free) metagenomics approaches are well suited for the detection of pathogens of different phylogenetic affiliations including viral and bacterial pathogens (e.g., Frank et al. 2011; Hanke et al. 2017; Rubbenstroth et al. 2019). In recent years, also infections caused by parasites (including asymptomatic intestinal infections caused by protists and helminths) have been detected using metagenomics (e.g., Kawai et al. 2012; Gao et al. 2016; Schneeberger et al. 2016). The detection of parasite signature sequences in metagenomics datasets can be improved using RNA instead of DNA, as shown for enteric protists in faecal samples (Wylezich et al. 2019). Indeed, the very large number of ribosomal reference sequences present in public databases allows the extraction and taxonomic assignments of ribosomal sequences from metagenomics datasets. In contrast, fully sequenced and well-annotated reference genomes are still lacking for many parasites, limiting the use of DNA-based sequencing and reference mapping approaches (e.g., Marzano et al. 2017; Stensvold and van der Giezen 2018). Another benefit of the untargeted metagenomics approach is that no prior decision is necessary for which pathogen to screen, i.e., which specific test system needs to be applied, since sequences derived from all potential pathogens will be present in the metagenomics dataset. This saves time and money by avoiding application of many laborious diagnostic techniques successively until a suspicion can be confirmed.

In a recent proof-of-concept study, we demonstrated the applicability of RNA metagenomics for parasite detection using faecal samples that were not pre-diagnosed for any enteric eukaryote (Wylezich et al. 2019). In the present follow-up study, we investigated pre-diagnosed samples with the same approach to confirm the original diagnosis made with conventional methods (microscopy, PCR) and to provide a more comprehensive picture of the non-host eukaryotes in the samples.

## Materials and methods

### Investigated sample materials

Diagnostic stool samples from different archive collections were included in this study: (1) Ethanol-fixed parasite-containing stool samples were obtained from the Medical University of Vienna, Austria (MUV samples). The samples were pre-diagnosed by light microscopy after sodium acetate-acetic acid-ethanol concentration. *Entamoeba*-positive samples were further differentiated at the species level by real-time PCR (Blessmann et al. 2002). The Friedrich Loeffler Institut (FLI) was provided with the samples including the results of the pre-diagnoses. (2) Stool samples were pre-diagnosed for protists and helminths using light microscopy and Lugol staining, and further screened by specific PCR assays for detection of *Ascaris, Giardia, Blastocystis* and *Entamoeba* (compare Table 1) at the Istituto Superiore di Sanità, Roma, Italy (ISS). To simulate an proficiency test for the metagenomics workflow, the ISS samples were sent frozen to the FLI without providing the results of the pre-diagnoses. Beside faecal material, (3) frozen samples of wild boar tissue infected with *Trichinella*, *Fasciola* and the lungworm *Dictyocaulus viviparus* were obtained from the official veterinary service of Western Pomerania, Anklam, Germany. Those veterinary-diagnostic (VD) samples were microscopically checked before transfer to the FLI and the diagnoses were provided with the material. Upon arrival at FLI, all samples were stored deeply frozen (−80 °C) until further processing.

**Table 1:**
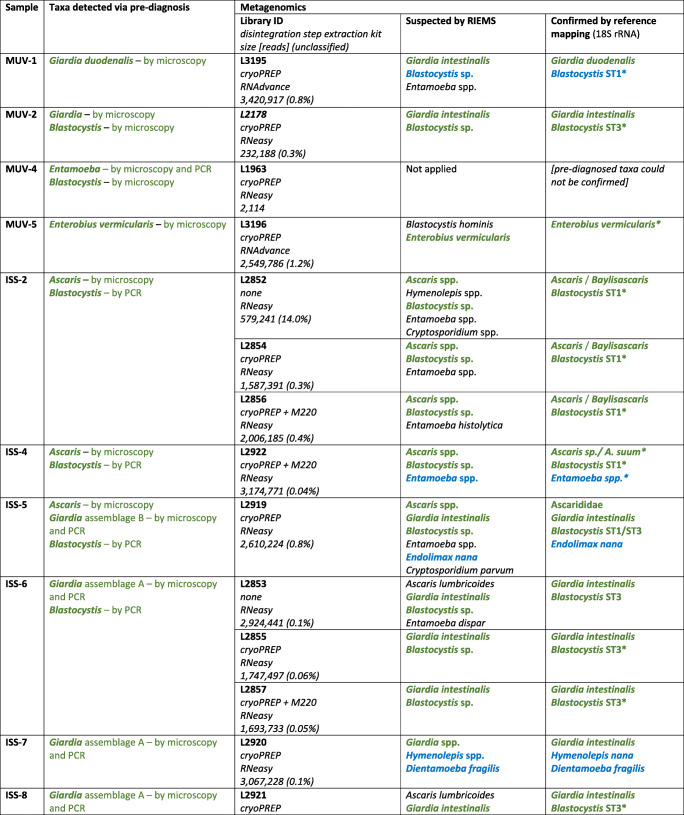

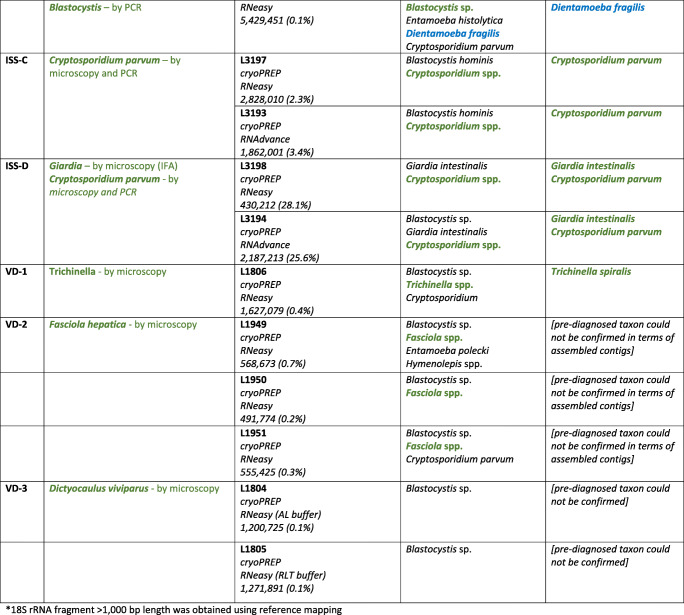
Samples investigated in the present study, pre-diagnosis and results obtained using metagenomics sequencing. MUV and ISS samples are stool samples; VD samples represent tissue samples. Taxa detected in the pre-diagnosis and taxa confirmed using metagenomics are shown in green, respectively. Taxa that were additionally found using metagenomics although not pre-diagnosed are shown in blue

### Metagenomics sequencing

Ethanol-fixed samples were centrifuged (12,000 rpm, 5 min, 4 °C) to remove ethanol, and the pellets were washed three times using 1 ml of 1 × TE buffer by mixing and subsequent centrifugation. The washed and re-suspended pellets (for MUV samples), the thawed ISS samples and pieces of the VD samples were treated with the cryoPREP (CP) instrument (Covaris) for disintegration as described (Wylezich et al. 2018). For two samples (ISS-2 and ISS-6), we additionally extracted RNA from subsamples without prior CP treatment and with combined treatment of CP plus Covaris M220 Focused-ultrasonicator (75 W, 1.5 min 7 °C). RNA was extracted in all cases, typically with the RNeasy Mini kit (Qiagen, Hilden, Germany). For two samples (ISS-C and ISS-D), disintegrated subsamples were additionally extracted using the Agencourt RNAdvance Tissue Kit (Beckman Coulter), following the manufacturer’s instructions (Table 1). Libraries were prepared from the RNA as described by Wylezich et al. (2018), and sequenced on the Ion Torrent S5XL platform.

### Detection of parasite signatures in metagenomics datasets

Generated metagenomics datasets were analysed using a combination of the RIEMS tool (Scheuch et al. 2015) and reference mapping against 18S rRNA gene sequences of suspected candidates using the Genome Sequencer software suite (versions 2.6; Roche) as described by Wylezich et al. (2019). Accession numbers of the sequences used as references are included in the **Supplementary Table S1**. Different identity thresholds (95–99%) and a minimum overlap length of reads of 95% were applied for reference mapping. The obtained 18S rRNA sequences were verified by Blast analysis (https://blast.ncbi.nlm.nih.gov/Blast.cgi?PROGRAM=blastn&PAGE_TYPE=BlastSearch&LINK_LOC=blasthome). Whereas for the proof-of-concept study (Wylezich et al. 2019) only nearly complete 18S rRNA sequences extracted from metagenomics datasets were rated as positive, in the present study, no minimum fragment length of the obtained contigs was presupposed for detection but instead each single read was considered positive if it was a specific hit.

The 18S rRNA sequences generated within this study were submitted to GenBank when the fragments were longer than 1000 base pairs (see **Supplementary Table S1**). They are accessible under the accession numbers MN914072-MN914086.

## Results and discussion

The 18S rRNA gene and other sequences of the ribosomal RNA cluster have been a preferential target for diagnostics of parasites (Kounosu et al. 2019, and references therein). However, primers used for diagnosis might miss certain parasite taxa or lead to cross-amplification of the (also eukaryotic) host or sample background, or generate short fragments not sufficient for a reliable phylogenetic assignment. Kounosu et al. (2019) evaluated several primer sets for 18S and 28S rRNA amplification and highlighted difficulties in identifying primers that enable synchronous detection of all eukaryotes, including parasites, in a single PCR. In particular, unicellular parasites were often not detected and received short amplicons were unsuited for an accurate taxonomic resolution (Kounosu et al. 2019). In the present study, we exploited the abundance of ribosomal sequences in metagenomics datasets obtained using RNA as starting material. The use of shotgun sequencing of total RNA, instead of DNA, should also positively shift the detection limit of PCR-diagnostics, due to the higher abundance of 18S rRNA transcripts compared to the corresponding coding genes (DNA), as recently demonstrated for the protistan parasite *Babesia* (Hanron et al. 2017).

### General performance of the untargeted metagenomics workflow

An overview of samples and associated results is given in Table 1. A detailed overview of used conditions (e.g. identity thresholds), resulting contigs and their identity to sequenced taxa from databases is provided as **Supplementary Table S1**. Overall, pre-diagnosed taxa were confirmed in all cases with the exception of samples MUV-4, VD-2 and VD-3. Concerning sample MUV-4, a very poor library could be constructed from the low amount of extracted RNA only delivering a minute sequence dataset (2114 reads). The fixation of the MUV-4 sample might have had a negative impact on the recovery of RNA. The resulting dataset was therefore too small for a successful detection of the taxa. Since helminths can be distributed very unevenly in the host tissue and their eggs in stool samples, respectively, the reason for failure with sample VD-3 could be the use of a disadvantageous subsample not containing any specimen of the pre-diagnosed taxa. This might also be true for VD-2. The dataset contained only a few reads of the liver fluke (*Fasciola hepatica* was found via RIEMS analysis, Table 1), which could not be assembled into contigs. For all other samples, pre-diagnosed parasites as well as additional protists were detected via untargeted metagenomics with yields between 0.00004% (for *Blastocystis* ST1 in sample ISS-5) and 15.7% (for *Cryptosporidium parvum* in sample ISS-C; **Supplementary Table S1**).

For two samples (ISS-2 and ISS-6), sample processing with disintegration (CP and CP + M220 Focused-ultrasonicator) and without disintegration was compared. For sample ISS-2, the detected number of reads from *Blastocystis* and *Ascaris* was higher without disintegration than with any disintegration. In these datasets, the number of parasite reads is especially influenced by shifts of the detected bacterial read numbers. As already shown, the application of a disintegration step may result in a higher representation of nucleic acids from specific certain bacteria (Wylezich et al. 2018). Regarding sample ISS-6, no difference in the number of parasite reads was found, yet inclusion of a disintegration step (CP and CP + M220 Focused-ultrasonicator) resulted in longer contigs compared to those obtained without prior disintegration (for *Blastocystis*). This is mainly caused by the amount of unclassified reads (Table 1) that was higher in datasets generated without prior disintegration compared with datasets that were generated with any disintegration (datasets of samples ISS-2 and ISS-6; Table 1). This negative impact in terms of unclassified reads in datasets generated without disintegration step has already been documented (Wylezich et al. 2018). While in the present cases, the disintegration resulted not always in an obviously better performance in terms of the parasite read amount, we recommend including this step to obtain high quality datasets and especially for detecting protistan cysts and/or helminth eggs.

With respect to the RNA extraction kits used, we could detect higher parasite read numbers for both samples ISS-C and ISS-D (*Cryptosporidium* spp.*, Giardia* spp.; **Supplementary Table S1**) extracted with the RNeasy Mini Kit compared to the datasets of the same samples extracted with the Agencourt RNAdvance Tissue Kit. Although the results for the two samples rather support the use of the RNeasy Mini Kit, the selection of the optimal kit needs to be investigated in a systematic validation and might depend on the sample type.

### Difficulties of the detection of *Giardia*

*Giardia* is the most commonly detected gut flagellate, worldwide, and can cause infections in humans and animals. The infective dose is known to be particularly low, already a few cysts can result in infection. In the present study, only one or two reads were found in microscopically *Giardia*-positive samples when using the 18S rRNA (M54878, AF006676, HQ179642) as reference sequence. When using a full genome sequence (UZAE01000001) for reference mapping, we found that most of the detected reads match with the rRNA tandem repeat unit but not necessarily with the 18S rRNA gene. Therefore, we re-run the reference mapping using the ribosomal tandem repeat (X52949) as reference, which resulted in a better mapping outcome though the read amount was still very low (<0.0009%) and reads mostly belonged to the 28S rRNA gene that is longer than the 18S rRNA gene. The ribosomal sequences of *Giardia* exhibit a very high GC content (about 74%, Wang et al. 2011) in contrast to the *Giardia* genome (about 46%, Smith et al. 1998). However, since genome sequences of *Giardia* were not overrepresented in the datasets over ribosomal sequences, we exclude a GC-shift of sequences that can be caused by some library preparation kits (Grützke et al. 2019). A possible explanation for the low recovery of *Giardia* reads can be a low number of *Giardia* cysts in the samples. Unfortunately, we have no quantitative data for the samples to verify this possibility. Also the storage conditions might have been suboptimal for these samples.

### Storage of samples

Basically, metagenomics sequencing enables to describe the sample complexity in terms of a sequence dataset, provided optimal storage of the sample to preserve its original state. Basically, fresh or deeply frozen material is well suited for metagenomics, especially when working with RNA. In the present study, samples were from the archives of diagnostic laboratories and not originally intended for metagenomics sequencing. Therefore, storage conditions might have been suboptimal, e.g., the cold chain was not fully maintained, and this may have caused loss of phylogenetic signals in terms of molecules for certain parasites. Ethanol fixation might be an alternative as shown here with the MUV samples when a freezer is not or not immediately available. However, one of the ethanol-fixed samples (MUV-4) resulted in a very poor library delivering only a small dataset.

### Additional findings besides the pre-diagnosed taxa

*Blastocystis* was confirmed in all pre-diagnosed cases and additionally found in a sample that had been pre-screened only by light microscopy but not by PCR indicating a low *Blastocystis* density in the respective sample (MUV-1). We detected additional species using the metagenomics approach. Since these taxa cause no severe diseases, according to the current state of knowledge, they are typically not included in routine diagnostics. For example, *Endolimax nana* belongs to the family Entamoebidae and is often observed in faecal samples but, due to its non-pathogenic nature, it is typically not reported. Likewise, enteric infections with *Hymenolepis nana* and *Dientamoeba fragilis* are typically considered benign, although controversy persists regarding their pathogenic potential. For example, *H. nana* was identified as causative agent in a case of invasive cestodiasis in an immunocompromised patient with a spread of the infection to an ectopic location (Olson et al. 2003). In terms of diagnostics, *D. fragilis* requires specific stainings or at least concentration methods and the analysis of fresh samples to observe the relevant morphological features. PCR diagnostics is sometimes applied for this parasite but bears the risk of false-positive results (Intra et al. 2019 and references therein). In the present study, the species was readily detected in metagenomics datasets.

Some implausible findings obtained with the RIEMS tool (e.g. *Blastocystis* in the VD tissue samples) are false-positive hits based on misassignments due to not correctly curated genomes in public databases or the lack of suitable reference genomes as already discussed in detail (Wylezich et al. 2019). For example, recently published sequences attributed to *Blastocystis* sp. (accession numbers MN339606, MK782501, MK782521) occurred in some Blast analyses as closest hits to the query sequence. However, the mentioned sequences are closely related to Dipodascaceae (Ascomycota) and do not show any close relationship to *Blastocystis* species (e.g. ST1, KY610205).

The aforementioned taxa might be often overlooked, at least when only present in low densities, and do therefore not contribute to the overall picture of a sample and are not included in the interpretation of the symptoms. For unresolved cases of intestinal disorders, however, it might be advantageous to get a more comprehensive picture of the individual gut microbiome allowing the interpretation of all comprised opportunistic agents and their mutual effects. In relation to this, a further benefit of the present method should be mentioned; it allows the parallel detection of prokaryotic taxa based on 16S and 23S rRNA without any primer bias. Another important and growing field of application for metagenomics-based diagnostics is the screening of organs and donor blood before transplantation or transfusion, respectively, as also asymptomatic, opportunistic pathogens may have serious consequences for the immunocompromised recipients (Hanron et al. 2017).

### Concluding remarks

Altogether, the application of untargeted RNA metagenomics sequencing for diagnostic samples was promising in the present study, especially since it can be used for the parallel identification of different unrelated taxa. In addition, it provides a more complete picture on protists that are often not included in routine diagnostics (e.g., *Dientamoeba fragilis,* “non-pathogenic” intestinal amoebae). Untargeted metagenomics sequencing is therefore applicable as one-serves-all pathogen detection tool swiftly providing comprehensive information of the sample. We recommend using it as a snapshot approach especially in cases with a difficult diagnosis because of contradictory symptoms or for infections in immunocompromised individuals. However, reasonable care has to be taken with the analysis of the obtained results using current public databases.

## Electronic supplementary material

ESM 1 (DOCX 38 kb)
