# Supplementary material for: Untargeted metagenomics shows a reliable performance for synchronous detection of parasites
Source: Parasitol Res. 2020 Jun 26;119(8):2623–9. doi: 10.1007/s00436-020-06754-9 (PMC7366571; doi:10.1007/s00436-020-06754-9)
Supplement: Supplementary file 1 — (DOCX 38 kb) [file 436_2020_6754_MOESM1_ESM.docx]

**Supplementary Table S1** Details of samples investigated in the present study, pre-diagnosis and results of metagenomics sequencing. MUV and ISS samples are stool samples; VD samples represent tissue samples. For detected taxa, the contig number, length and read number per contig is given as well as the closest blastn hit. Contigs marked with an asterisk (column “contig length”) are deposited in GenBank under the accession numbers MN914072-MN914086. Abbreviations; -mi, minimal identity of reads to each other used for reference mapping; -ml, minimal overlapping length of reads used for reference mapping.

| **sample** | **pre-diagnosis** | **library ID** | **reference for mapping, accession number** | **-mi**  **[%]** | **-ml**  **[%]** | **contig no.** | **contig length** | **read number** | **closest blastn hits to sequences in GenBank** | **identity [%]** | **amount**  **[%]** |
| --- | --- | --- | --- | --- | --- | --- | --- | --- | --- | --- | --- |
| MUV-1 | Giardia duodenalis | L3195 | Giardia ribosomal RNA tandem repeat, X52949 | 95 | 95 | 1 | 459 | 6 | Giardia intestinalis (28S rRNA) | 97.7 | 0.0002 |
|  |  |  | Blastocystis ST1 18S rRNA, AB070989 | 98 | 95 | 1 | *1678 | 67 | Blastocystis ST1 | 99.6 | 0.002 |
| MUV-2 | Giardia | L2178 | Giardia ribosomal RNA tandem repeat, X52949 | 98 | 95 | 1 | 345 | 2 | Giardia intestinalis (28S rRNA) | 98.8 | 0.0009 |
|  | Blastocystis |  | Blastocystis ST3 18S rRNA, AB107963 | 98 | 95 | 1 | *1715 | 91 | Blastocystis ST3 | 100,0 | 0.04 |
| MUV-4 | Entamoeba | L1963 | Entamoeba histolytica 18S rRNA, X56991 | 98 | 95 | - |  |  |  |  |  |
|  | Blastocystis |  | Blastocystis ST3, AB107963; ST1, AB070989 | 98 | 95 | - |  |  |  |  |  |
| MUV-5 | Enterobius vermicularis | L3196 | Enterobuis vermicularis 18S rRNA, JF934731 | 98 | 95 | 1 | *1782 | 237 | Enterobius vermicularis | 99.9 | 0.009 |
| ISS-2 | Ascaria | L2852 | Ascaris sp. 18S rRNA, JN256985 | 98 | 95 | 1 | 479 | 4 | Ascaris | 99.4 | 0.002 |
|  |  |  |  |  |  | 2 | 264 | 2 | Ascaris | 99.6 |  |
|  |  |  |  |  |  | 3 | 292 | 8 | Ascaris | 99.3 |  |
|  |  |  | Blastocystis ST1 18S rRNA, AB070989 | 98 | 95 | 1 | *1665 | 219 | Blastocystis ST1 | 99.8 | 0.04 |
|  |  | L2854 | Ascaris sp. 18S rRNA, JN256985 | 98 | 95 | 1 | 248 | 4 | Ascaris | 100,0 | 0.0005 |
|  |  |  |  |  |  | 2 | 271 | 1 | Ascaris | 99.6 |  |
|  |  |  |  |  |  | 3 | 495 | 3 | Ascaris | 99.8 |  |
|  |  |  | Blastocystis ST1 18S rRNA, AB070989 | 96 | 95 | 1 | 1683 | 49 | Blastocytis ST1 | 99.4 | 0.003 |
|  |  | L2856 | Ascaris sp. 18S rRNA, JN256985 | 98 | 95 | 1 | 219 | 11 | Ascaris | 99.5 | 0.001 |
|  |  |  |  |  |  | 2 | 364 | 12 | Ascaris | 100,0 |  |
|  |  |  |  |  |  | 3 | 158 | 1 | Ascaris | 100,0 |  |
|  |  |  |  |  |  | 4 | 174 | 4 | Ascaris | 100,0 |  |
|  |  |  | Blastocystis ST1 18S rRNA, AB070989 | 98 | 95 | 1 | *1614 | 61 | Blastocystis ST1 | 99.9 | 0.003 |
| ISS-4 | Ascaris | L2922 | Ascaris sp. 18S rRNA, JN256985 | 98 | 95 | 1 | *1469 | 57 | Ascaris | 99.9 | 0.002 |
|  |  |  | Blastocystis ST1 18S rRNA, AB070989 | 96 | 95 | 1 | 460 | 34 | Blastocystis ST1 | 100,0 | 0.003 |
|  |  |  |  |  |  | 2 | *1235 | 54 | Blastocytis ST1 | 99.4 |  |
|  |  |  | Entamoeba coli 18S rRNA, AF149915 | 98 | 95 | 1 | 771 | 1163 | Entamoeba coli | 99.9 | 0.07 |
|  |  |  |  |  |  | 2 | *1174 | 963 | Entamoeba coli | 99.8 |  |
| ISS-5 | Ascaris | L2919 | Ascaris sp. 18S rRNA, JN256985 | 98 | 95 | 1 | 185 | 2 | Ascaris/Toxocara | 100,0 | 0.0002 |
|  |  |  |  |  |  | 2 | 216 | 2 | Ascaris/Toxocara | 99.1 |  |
|  |  |  |  |  |  | 3 | 120 | 2 | Toxascaris | 100,0 |  |
|  | Giardia assemblage B |  | Giardia ribosomal RNA tandem repeat, X52949 | 98 | 95 | 1 | 119 | 4 | Giardia intestinalis (28S rRNA) | 98.3 | 0.0002 |
|  |  |  | Blastocystis ST1 18S rRNA, AB070989 | 98 | 95 | 1 | 290 | 1 | Blastocystis ST1 | 99,0 | 0.00004 |
|  |  |  | Blastocystis ST3 18S rRNA, AB107963 | 98 | 95 | 1 | 206 | 1 | Blastocystis ST3 | 100,0 | 0.0004 |
|  |  |  |  |  |  | 2 | 186 | 6 | Blastocystis ST3 | 98.4 |  |
|  |  |  |  |  |  | 3 | 311 | 3 | Blastocystis ST3 | 100,0 |  |
|  |  |  | Endolimax nana 18S rRNA, AF149916 | 98 | 95 | 1 | 296 | 1 | Endolimax nana | 99,0 | 0.0002 |
|  |  |  |  |  |  | 2 | 103 | 1 | Endolimax nana | 100,0 |  |
|  |  |  |  |  |  | 3 | 315 | 3 | Endolimax nana | 99.4 |  |
| ISS-6 | Giardia assemblage B | L2853 | Giardia ribosomal RNA tandem repeat, X52949 | 98 | 95 | 1 | 106 | 1 | Giardia intestinalis (28S rRNA) | 99.1 | 0.0003 |
|  |  |  |  |  |  | 2 | 150 | 1 | Giardia intestinalis (28S rRNA) | 98.7 |  |
|  |  |  |  |  |  | 3 | 267 | 4 | Giardia intestinalis (28S rRNA) | 100,0 |  |
|  |  |  |  |  |  | 4 | 275 | 2 | Giardia intestinalis (28S rRNA) | 98.6 |  |
|  | Blastocystis ST3 |  | Blastocystis ST3 18S rRNA, AB107963 | 98 | 95 | 1 | 525 | 23 | Blastocystis ST3 | 100,0 | 0.002 |
|  |  |  |  |  |  | 2 | 719 | 25 | Blastocystis ST3 | 99.9 |  |
|  | Giardia assemblage B | L2855 | Giardia ribosomal RNA tandem repeat, X52949 | 98 | 95 | 1 | 221 | 3 | Giardia intestinalis (5.8S rRNA) | 99.1 | 0.0006 |
|  |  |  |  |  |  | 2 | 101 | 2 | Giardia intestinalis (28S rRNA) | 100,0 |  |
|  |  |  |  |  |  | 3 | 487 | 5 | Giardia intestinalis (28S rRNA) | 99.4 |  |
|  | Blastocystis ST3 |  | Blastocystis ST3 18S rRNA, AB107963 | 98 | 95 | 1 | *1592 | 40 | Blastocystis ST3 | 99.6 | 0.002 |
|  | Giardia assemblage B | L2857 | Giardia ribosomal RNA tandem repeat, X52949 | 98 | 95 | 1 | 145 | 1 | Giardia intestinalis (5.8S rRNA) | 99.3 | 0.0003 |
|  |  |  |  |  |  | 2 | 332 | 2 | Giardia intestinalis (28S rRNA) | 99.7 |  |
|  |  |  |  |  |  | 3 | 110 | 2 | Giardia intestinalis (28S rRNA) | 100,0 |  |
|  | Blastocystis ST3 |  | Blastocystis ST3 18S AB107963 | 98 | 95 | 1 | *1602 | 30 | Blastocystis ST3 | 99.6 | 0.002 |
| ISS-7 | Giardia assemblage A | L2920 | Giardia ribosomal RNA tandem repeat, X52949 | 98 | 95 | 1 | 235 | 1 | Giardia intestinalis (18S rRNA) | 100,0 | 0.0004 |
|  |  |  |  |  |  | 2 | 118 | 3 | Giardia intestinalis (5.8S rRNA) | 100,0 |  |
|  |  |  |  |  |  | 3 | 109 | 5 | Giardia intestinalis (5.8S rRNA) | 99.1 |  |
|  |  |  |  |  |  | 4 | 364 | 2 | Giardia intestinalis (28S rRNA) | 98.3 |  |
|  |  |  | Hymenolepis diminuta 18S rRNA, AF286983 | 98 | 95 | 1 | 391 | 6 | Hymenolepis nana | 100,0 | 0.0007 |
|  |  |  |  |  |  | 2 | 573 | 15 | Hymenolepis nana | 99.7 |  |
|  |  |  | Dientamoeba fragilis 18S rRNA, U37461 | 95 | 95 | 1 | 134 | 3 | Dientamoeba fragilis | 99.3 | 0.007 |
|  |  |  |  |  |  | 2 | 552 | 72 | Dientamoeba fragilis | 99.1 |  |
|  |  |  |  |  |  | 3 | 865 | 153 | Dientamoeba fragilis | 99.7 |  |
| ISS-8 | Giardia assemblage A | L2921 | Giardia ribosomal RNA tandem repeat, X52949 | 98 | 95 | 1 | 170 | 1 | Giardia intestinalis (28S rRNA) | 99.4 | 0.0001 |
|  |  |  |  |  |  | 2 | 124 | 1 | Giardia intestinalis (5.8S rRNA) | 99.2 |  |
|  |  |  |  |  |  | 3 | 157 | 1 | Giardia intestinalis (28S rRNA) | 99.4 |  |
|  |  |  |  |  |  | 4 | 121 | 1 | Giardia intestinalis (28S rRNA) | 99.2 |  |
|  |  |  |  |  |  | 5 | 342 | 3 | Giardia intestinalis (28S rRNA) | 98.8 |  |
|  |  |  |  |  |  | 6 | 323 | 1 | Giardia intestinalis (28S rRNA) | 99.4 |  |
|  | Blastocystis ST3 |  | Blastocystis ST3 18S rRNA, AB107963 | 98 | 95 | 1 | *1729 | 1302 | Blastocystis ST3 | 99.9 | 0.02 |
|  |  |  | Dientamoeba fragilis 18S rRNA, U37461 | 95 | 95 | 1 | *1620 | 392 | Dientamoeba fragilis | 99.5 | 0.007 |
| ISS-C | Cryptosporidium parvum | L3197 | Cryptpsporidium parvum 18S rRNA, AF108865 | 98 | 95 | 1 | *1748 | 445410 | Cryptpsporidium parvum | 99.9 | 15.7 |
|  |  | L3193 | Cryptpsporidium parvum 18S rRNA, AF108865 | 98 | 95 | 1 | 1748 | 242408 | Cryptpsporidium parvum | 99.9 | 13.0 |
| ISS-D | Cryptosporidium parvum | L3198 | Cryptpsporidium parvum 18S rRNA, AF108865 | 98 | 95 | 1 | *1748 | 4861 | Cryptpsporidium parvum | 99.9 | 1.1 |
|  | Giardia |  | Giardia ribosomal RNA tandem repeat, X52949 | 98 | 95 | 1 | 399 | 2 | Giardia intestinalis (28S rRNA) | 99.3 | 0.0005 |
|  | Cryptosporidium parvum | L3194 | Cryptpsporidium parvum 18S rRNA, AF108865 | 98 | 95 | 1 | 1748 | 9397 | Cryptpsporidium parvum | 99.9 | 0.4 |
|  | Giardia |  | Giardia ribosomal RNA tandem repeat, X52949 | 98 | 95 | 1 | 105 | 1 | Giardia intestinalis (28S rRNA) | 98.1 | 0.00005 |
| VD-1 | Trichinella | L1806 | Trichinella sp. 18S rRNA, AY851260 | 98 | 95 | 1 | *1784 | 1048 | Trichinella spiralis | 98.4 | 0.06 |
| VD-2 | Fasciola hepatica | L1949 | Fasciola hepatica 18S rRNA, MF077355 | 98 | 95 | - |  |  |  |  |  |
|  |  | L1950 | Fasciola hepatica 18S rRNA, MF077355 | 98 | 95 | - |  |  |  |  |  |
|  |  | L1951 | Fasciola hepatica 18S rRNA, MF077355 | 98 | 95 | - |  |  |  |  |  |
| VD-3 | Dictyocaulus viviparus | L1804 | Dictyocaulus viviparus 18S rRNA, AJ920361 | 98 | 95 | - |  |  |  |  |  |
|  |  | L1805 | Dictyocaulus viviparus 18S rRNA, AJ920361 | 98 | 95 | - |  |  |  |  |  |
